# Retraction of: Corrosion-Resistant Multi-principal Ni_60_Cr_15_Co_15_Ti_5_Al_2.5_Nb_2.5_ Alloy: Loss of Passivation (LOP) and Recovery of Passivation (ROP) upon Heat Treatment

**DOI:** 10.1021/acsomega.6c09779

**Published:** 2026-09-03

**Authors:** Virgilio Pereira Ricci, Emerson Cortez Gallego Campos, Victor Hugo Mafra Monfredo Ferreira, Rafael Magalhães Triani, Guilherme Zepon, Francisco Gil Coury, Eric Marchezini Mazzer, Guilherme Yuuki Koga

The authors retract this article (DOI: 10.1021/acsomega.5c06996) due to errors in the structural characterization section related to phase identification and the associated discussion in the text and figures. Although the manuscript primarily focuses on corrosion behavior, phase identification in this complex multiprincipal alloy system requires more in-depth characterization to ensure clarity and accuracy in the interpretation of the results. The corrected text and figures are being published concurrently with this retraction notice, in an article entitled “Corrosion Resistant Multi-Principal Ni_60_Cr_15_Co_15_Ti_5_Al_2.5_Nb_2.5_ Alloy: Loss of Passivation (LOP) and Recovery of Passivation (ROP) Upon Heat Treatment”, DOI: 10.1021/acsomega.6c08640.

The original article was published on January 23, 2026 and retracted on September 3, 2026.

